# Investigation of a Rare Occurrence of a Diatomaceous Coating of the Cotswold Weir Wall on the Condamine River, Australia

**DOI:** 10.3390/plants14030332

**Published:** 2025-01-23

**Authors:** John P. Thompson, John Standley, Rachel C. Hancock

**Affiliations:** 1 Centre for Crop Health, Institute for Life Sciences and the Environment, University of Southern Queensland, Toowoomba, QLD 4350, Australia; 2 Condamine-Balonne Water Committee, Toowoomba, QLD 4350, Australia; john.standley@bigpond.com; 3 Central Analytical Research Facility, Institute for Future Environments, Queensland University of Technology, Brisbane, QLD 4001, Australia; rje001@gmail.com

**Keywords:** diatoms, weir wall, Australian river, Murray–Darling River system, water quality

## Abstract

A white encrustation of the Cotswold Weir wall in the lower reaches of the Condamine River, a tributary of the Murray–Darling River system in semi-arid Australia, was investigated following community concern that it indicated health risks from an unknown substance in the water resulting from mining and agricultural enterprises in the catchment. The vitreous white surface consisted of closely packed frustules of diatoms, observed by scanning electron microscopy, with an underlying layer of clay particles and dried filamentous green algae. Pennate diatoms identified in the white encrustation were the benthic species *Nitzschia palea* (predominant), *Eolimna subminiscula*, *Craticula* aff. *cuspidata*, *Navicula viridula* var. *rostellata*, and *Luticola mutica*. The centric diatom species *Melosira varians* was also present as filamentous chains of cylindrical frustules among the aggregated pennate diatom frustules. The encrustation was the remains of a periphyton (biofilm) of diatoms and green algae that had developed during protracted stream flow over the weir wall following record flooding. A dry period had resulted in the death of the diatoms and exposure of their aggregated siliceous frustules as a vitreous white coating. All diatom species identified are considered tolerant of eutrophic and mildly saline conditions. Chemical analyses of water from the Cotswold Weir, compared to long-term records, revealed higher salinity, with changes from March when the river was flowing to September when the white coating was noted, in electrical conductivity (299 to 461 µS/cm), and in sodium (26 to 43 mg/L) and chloride (26 to 75 mg/L) concentrations, respectively. Total nitrogen (0.82 to 1.6 mg/L) and total phosphorus (0.24 to 0.094 mg/L) were at mesotrophic and eutrophic concentrations, respectively, together with substantial dissolved silica concentrations (18 to 11 mg/L). Atomic ratios for total nitrogen/total phosphorus (7.6 to 37.6), nitrate-nitrogen/orthophosphate-phosphorus (2.3 to 274), and dissolved silica–silicon:orthophosphate-phosphorus (81.7 to 749) probably favoured diatoms over other photoautotrophs. While the diatomaceous encrustation indicated no health risks from the weir water, continued watch is required to avoid eutrophication and salinization of the river.

## 1. Introduction

**Figure 1:**
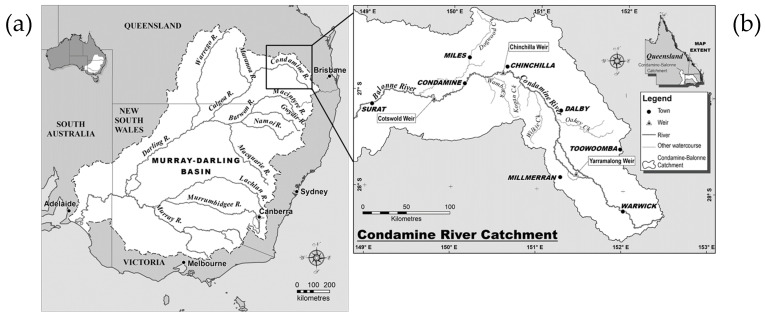
(**a**) The Murray–Darling River system showing the location of the tributary Condamine River in Queensland; (**b**) map of the Condamine–Balonne River system showing the location of Cotswold Weir.

The Murray–Darling is the longest river system in Australia passing through the states of Queensland, New South Wales, Victoria, and South Australia (Figure 1a). The Murray–Darling Basin is economically important contributing 40% of Australian agricultural production, as well as being of environmental, cultural, and recreational significance [1]. A major tributary of this river system in Queensland is the Condamine River (Figure 1b), which rises on Mount Superbus (28.2167° S, 152.4667° E) on the western side of the Great Dividing Range about 100 km south-west of Brisbane. The river rises at an elevation of 772 m and flows inland in a north-westerly, then westerly, direction for about 660 km. The river, with a catchment area of 13,292 km^2^, passes through the rich agricultural area known as the Darling Downs, noted for its fertile clay soils classified as Vertosols [2]. The Condamine River changes its name to Balonne River (at an elevation of 256 m) before reaching the township of Surat (27.1544° S, 149.0677° E).

The climate of the Condamine River catchment is subtropical, modified by an elevation of up to 500 m above sea level. Average monthly temperature is highest in January (28–33 °C) and lowest in July (17–19 °C). The average rainfall is ~800 mm/year near the source of the river, decreasing to ~600 mm/year further inland, while the average evaporation is 1600 to 2000 mm/year, making most of the area subhumid to semi-arid [3]. The rainfall is summer-dominant on average, but quite variable both seasonally and annually, with the catchment subject to periods of drought and occasional floods. Thus, conditions in the river cover extremes from fast flow of large volumes of water during floods, to low flow of small volumes or no flow during prolonged droughts, when the river can be reduced to a series of lagoons. A number of weirs have been built along the Condamine River to retain water for supply to towns and agricultural irrigation.

The Darling Downs area produces mainly dryland summer grain crops of sorghum (*Sorghum bicolor*) and mungbean (*Vigna radiata*), and winter crops of wheat (*Triticum aestivum*), barley (*Hordeum vulgare*), and chickpea (*Cicer arietinum*). Irrigated cotton (*Gossypium hirsutum*) and maize (*Zea mays*) are grown with water supplied from ring tanks that collect overland flow during periods of intense rainfall, as well as with water pumped from underground aquifers or directly from the Condamine River system. Animal industries, including beef cattle feedlots, are important components of primary production on the Darling Downs. Mining is another important industry on the Darling Downs with five coal mines, as well as coal seam gas wells [4].

There were record floods in the Condamine River in December 2010 and again in January 2011. During these two floods, the river reached respective peaks of 14.39 m and 12.41 m at the Chinchilla Weir (26.8000° S, 150.6528° E), and 15.25 m and 14.67 m at the township of Condamine (26.9258° S, 150.1363° E), where >8 m is classified as a major flood [5]. Subsequent to these flood events, there was a long period of continuous water flow in the Condamine River. In 2012, the Condamine–Balonne Water Committee commissioned a study of the Condamine–Balonne River system, to test possibly new levels of chemical contaminants of concern for public and river health [4]. On sampling of the water in Cotswold Weir (27.0866° S, 149.7866° E) (Figure 1b), between the townships of Condamine and Surat in early September 2012, a white coating was present on the concrete wall of the weir.

**Figure 2:**
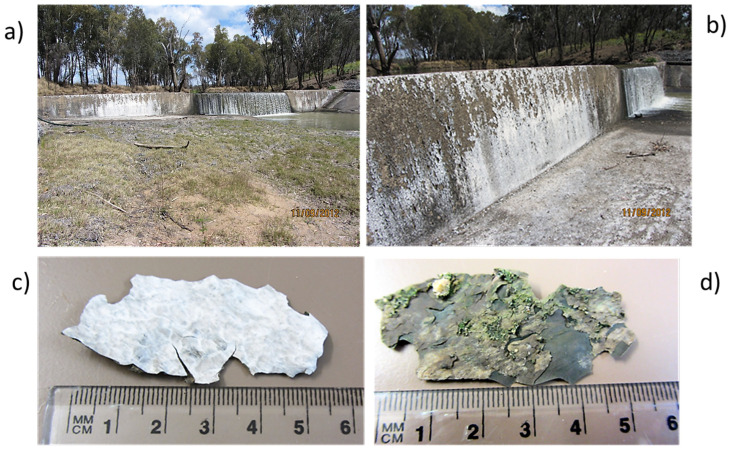
(**a**,**b**) Two views of the Cotswold Weir wall with a white substance coating the dry higher side walls and water flowing over the lower central section of the weir; (**c**) upper surface of a white flake from the wall; and (**d**) smooth dark grey underside of a flake with sections of green.

There had been a low weir at this site since 1990, which was then raised by 3.22 m to an overall height of 4.29 m in 1994 (K. Klaasen pers. comm.). At the time of sampling, there was a steady flow of water about 5 cm above the central part of the weir wall, but no flow over the higher side walls, which had a noticeable white encrustation (Figure 2a,b). The white material could be broken in flakes from the weir wall (Figure 2c,d).

Several articles appeared in the regional press about the white substance, which had also been noted on the Chinchilla Weir and along the banks of the Cotswold Weir pool. Concerns were expressed by members of the community about the unknown substance and its origin as well as any implications about whether the water was unsuitable for consumption by humans and livestock. Some people proposed that the substance was salt due to the entry of saline water into the river system from mining activities, while others proposed it was carbonate that had formed naturally and had not originated from any form of river pollution. Concerns were also heightened with the appearance of methane gas seeps in the Condamine River near Chinchilla [6] and that these seeps had altered the water in the river causing the white deposit. As several opinions for the origin of this previously unseen phenomenon of the white coating of the weir wall were put forward, we decided to investigate the nature of the white surface of flakes collected from the Cotswold Weir wall to learn whether it was biological or chemical in origin. The first author had the previous experience of investigating a white encrustation on the surface of a clay soil (Black Vertosol) [2] from a low area in a grain grower’s field (27.4461° S, 151.4349° E) on the Darling Downs, which had dried following prolonged wet weather. This white coating of the soil surface raised fears of rising salinity in the field, but measurements showed that the electrical conductivity of the soil was normal. Microscopic observations of scrapings of the white soil surface showed the presence of diatom frustules. Furthermore, illuminated incubation of pieces of the white-encrusted soil in water resulted in the growth of green algae and many pennate diatoms. Thus, in that instance, the growth and death of diatoms had left a white coating on the soil surface. This experience led us to consider diatoms as a possible cause of the white coating on the Cotswold Weir wall. Diatoms are microscopic algae (Division Bacillariophyta), which possess a cell wall composed of silica (frustules). They are common in water bodies both marine and freshwater and in damp terrestrial environments. When diatoms die, their accumulated siliceous frustules can appear as a white substance and large geological deposits are mined as diatomite or diatomaceous earth. 

Different diatom species may have different ecological requirements and tolerances. Consequently, they have been utilised as bioindicators of environmental conditions, particularly water quality. Such studies have included not only lakes and reservoirs likely to be heavily polluted in urban environments [7], but also rivers affected by eutrophication, sedimentation and salinization [8,9,10], including some rivers in semi-arid environments [11], similar to the environment of the Condamine River. Diatom growth in rivers in semi-arid environments is influenced by extreme variations in rainfall events affecting runoff from land into the river with attendant nutrient loads of silica, nitrogen and phosphorus that can stimulate diatom growth, followed by dry periods with no water flow that desiccates diatoms on exposed surfaces. 

The purpose of this paper is to report investigations into the nature of the white substance that coated the Cotswold Weir wall and the implications of the results for the ecological health of the Condamine River and for communities dependent upon water drawn from it. This is the first investigation to prove scientifically that the blooming of diatoms and their subsequent death and bleaching can extensively coat an important river structure with a white encrustation in semi-arid Queensland, thereby reducing community concerns about other potential causes, and relating this phenomenon to climatic and hydrological factors.

## 2. Results

### 2.1. Macroscopic and Microscopic Characteristics of the White Flakes

The collected flakes were up to 50 mm across and 200 µm thick. They were white on the top side and dark grey on the underside with adherent smaller flakes of green. When observed under a stereo microscope, the upper surface of the flakes appeared white and shiny with fine granular structure and a few flecks of dark colour. Where drops of dilute hydrochloric acid were placed on the upper white surface of flakes and observed under the stereo microscope, isolated bubbles formed slowly from within the flake indicating the presence of some carbonate, but there was no rapid effervescence from the white surface indicating that it was not principally composed of carbonates.

**Figure 3:**
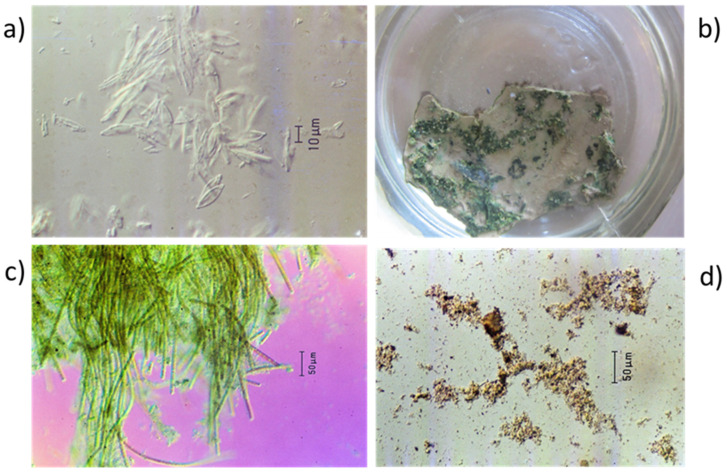
(**a**) Silica frustules of pennate diatoms seen in scrapings from the white upper surface of a dried flake of white encrustation when viewed by interference contrast microscopy (grey background); (**b**) appearance of green material on the upper surface of a flake after 4-day incubation in water in diffuse sunlight. Flake = 2 cm long; (**c**) filamentous, multicellular green algae from within a white flake after 4-day incubation in water in diffuse sunlight as seen by differential interference contrast microscopy (purple background); and (**d**) amorphous particulate material from the underside of a flake after 4-day incubation in water as seen by differential interference contrast microscopy (grey background).

In scrapings of the white surface mounted in water on microscope slides and observed, using differential interference contrast microscopy, the spindle-shaped frustules of dead diatoms (Order Pennales) about 20 µm long (Figure 3a) were evident at magnifications of ×400 and greater.

### 2.2. Growth of Algae from the Flakes During Illuminated Incubation in Water

After 24 h of incubation in water in diffuse sunlight, parts of the top surface of the flakes in all jars appeared green. The appearance of a flake after 4 days of incubation is shown in Figure 3b. In microscope slides made of the green material, a mat of filamentous green algae was seen under the microscope (Figure 3c). The algae appeared to be covered with an amorphous brown material. Amongst the filaments of the algae were diatom frustules, but no live diatoms were seen during incubation for up to 30 days.

Microscope slides prepared from the bottom of the flakes after incubation in water showed the presence of much particulate material, probably mainly clay particles and fragments of humified organic matter (Figure 3d).

### 2.3. Diatoms Forming the White Surface of the Flakes Revealed by Scanning Electron Microscopy

**Figure 4:**
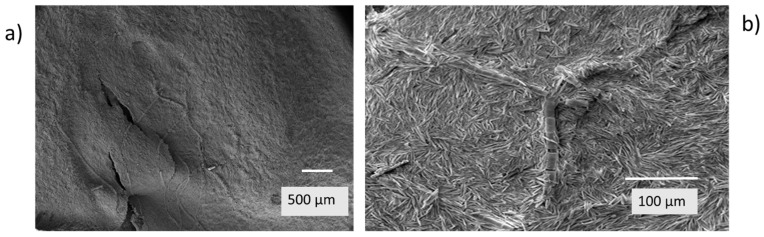
Electron micrograph overviews of the white surface of a flake exhibiting: (**a**) a finely granular surface composed of pennate diatoms with some ‘tracks’ of centric diatom chains; and (**b**) a view of a chain of cylindrical frustules of a centric diatom in a bed of pennate diatom frustules at higher magnification.

After diatom frustules were observed with light microscopy, further images of the undisturbed white surface of the flakes were obtained by scanning electron microscopy (SEM). From the electron micrographs, it was seen that the white surface was composed largely of the frustules of pennate diatoms closely packed together (Figure 4a,b) with frustules of some radially symmetric centric diatoms (order Centrales) forming filaments running through the aggregation of pennate diatom frustules.

**Figure 5:**
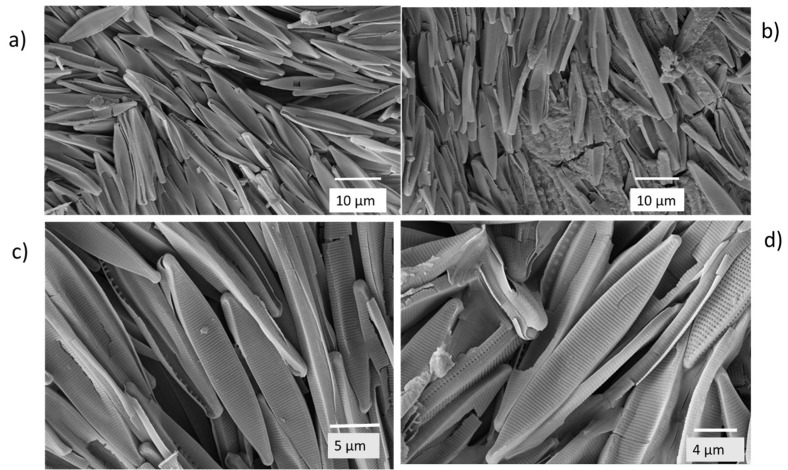
Electron micrograph views of (**a**) pennate diatom frustules forming sheets; (**b**) frustules intermingled with an underlay of fine amorphous material, probably clay; and (**c**,**d**) most common frustules were of *Nitzschia palea*.

The appearance of the surface at higher magnification was of closely packed frustules of pennate diatoms (Figure 5a) with an underlying amorphous material, probably clay particles, evident in some fields of view (Figure 5b). The predominant frustules were of *Nitzschia palea* (Figure 5c,d).

**Figure 6:**
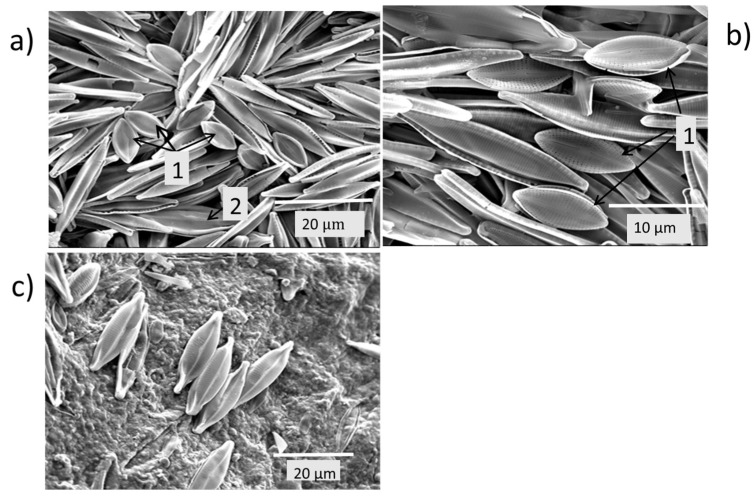
Electron micrographs of frustules of pennate diatoms in the white coating (**a**) mainly *Nitzschia palea* with 1 *Eolimna subminiscula*, and 2 *Craticula* aff. *cuspidata*; (**b**) 1 *Eolimna subminiscula* amongst *Nitzschia palea*; and (**c**) *Navicula viridula* var. *rostellata*.

Frustules of other species present were *Eolimna subminiscula* (Figure 6a,b), *Craticula* aff. *cuspidata* (Figure 6a), and *Navicula viridula* var. *rostellata* (Figure 6c).

**Figure 7:**
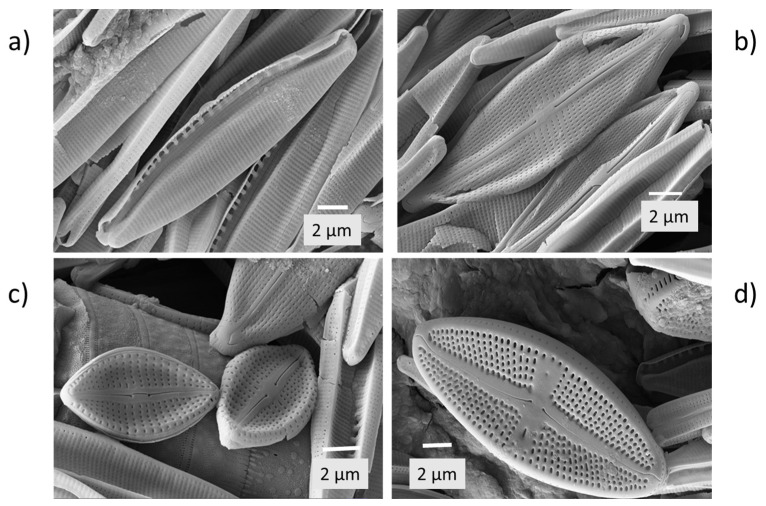
Electron micrographs of frustules of pennate diatom species present in the white encrustations (**a**) *Nitzschia palea*; (**b**,**c**) *Eolimna subminiscula*; and (**d**) *Luticola mutica*.

Examination of the predominant *Nitzschia palea* (Figure 5c,d; Figure 7a) revealed 35–36 striae per 10 µm length of the frustules, indicating it to be *Nitzschia palea* var. *palea* rather than *Nitzschia palea* var. *debilis*, which has ≥43 striae per 10 µm [12]. Other pennate diatom species identified from the higher magnification images were *Eolimna subminiscula* (Figure 7b,c) and *Luticola mutica* (Figure 7d).

**Figure 8:**
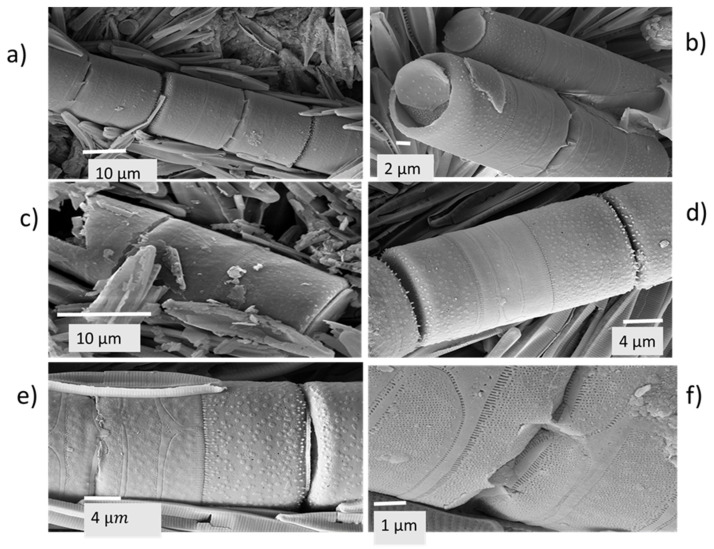
Electron micrographs at various magnifications of the chained frustules of the centric diatom *Melosira varians* present in the white encrustation: (**a**) segment of a chain of cylindrical frustules; (**b**) end view of a frustule; (**c**) chain of *M. varians* frustules embedded among pennate diatoms; (**d**) close view of a single frustule; (**e**) ornamentation on the frustule surface; (**f**) striae patterns of pores, and spines linking frustules.

Apart from these predominantly pennate diatoms, radially symmetric diatoms (order Centrales) appearing as filaments consisting of cylindrical frustules positioned end to end, were evident in some of the electron micrographs; with the species identified as *Melosira varians* (Figure 8a–f).

### 2.4. Preceding Water Flows over the Cotswold Weir

**Figure 9:**
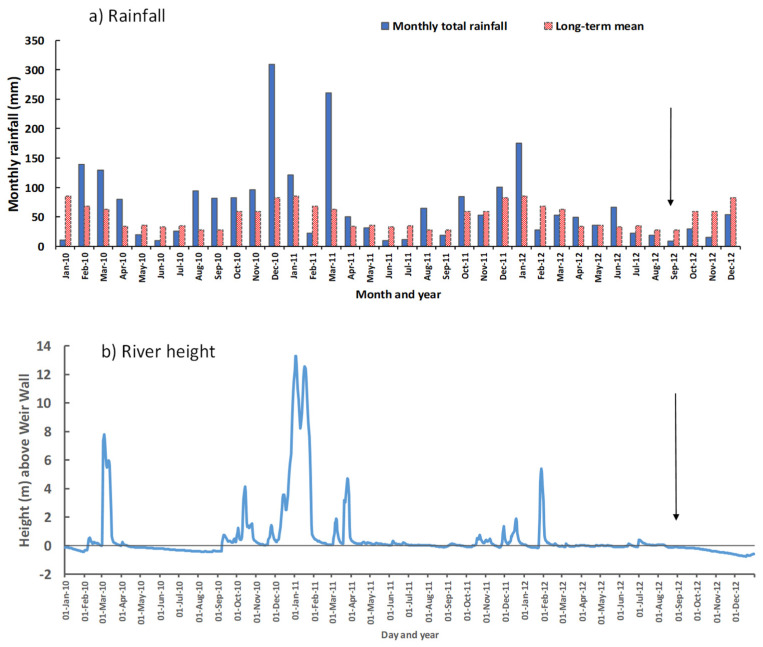
(**a**) Total monthly rainfall (mm) for the years 2010–2012 at the township of Condamine, compared with long-term means from 1937 to 2018 [5]; (**b**) daily height of the Condamine River above the Cotswold Weir side wall in 2010–2012 where a positive value indicates water flow over the wall at gauging station No. 422325A [13]. The side wall height is 4.5 m represented by a horizontal black line at 0 m. Vertical arrows indicate the date of sampling (11 September 2012) of the white encrustation on the weir wall.

There was record flooding in the Condamine River in late December 2010 and early January 2011, following extreme rainfall across the entire catchment. At the township of Condamine, situated upstream from the Cotswold Weir, the rainfall of 324 mm in December 2010 was the highest monthly total in 82 years of record keeping (Figure 9a). From the records of river heights [13], there was a flood in the river in March 2010 followed by no flow over the side wall of the Weir from 11 April to 8 September 2010 (Figure 9b). Subsequently, there were substantial flows leading up to peak river heights of 13.28 and 12.53 m above the side wall on 2 and 17 January 2011, respectively. For the period of 704 days from 10 September 2010 to 12 August 2012, there was continual flow over the lower central section of the wall, with flow over the side wall on 76% of days. From 12 August to 8 September 2012, when the samples were collected, there was no flow over the side wall.

### 2.5. Physical and Chemical Properties of the Water

**Figure 10:**
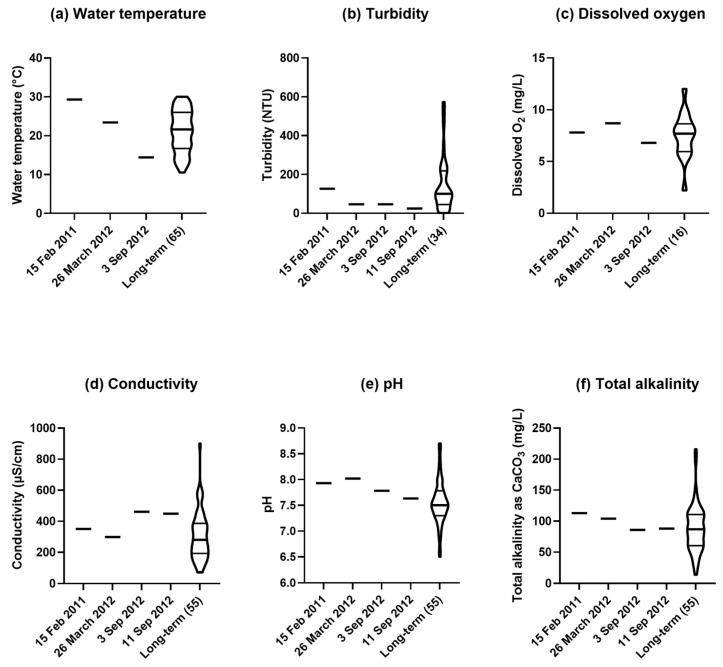
Physical and chemical properties of water samples from the Cotswold Weir for four sampling dates, relevant to when the white encrustation was collected on 11 September 2012, in comparison with long-term results for the period from March 1971 to August 2019 [13]: (**a**) water temperature; (**b**) turbidity; (**c**) dissolved oxygen; (**d**) electrical conductivity; (**e**) pH; and (**f**) total alkalinity. Each violin plot shows the smoothed frequency distribution of values for the trait of interest from long-term sampling, with the median indicated by the heavy horizontal line and first (lower) and third (upper) quartiles indicated by lighter horizontal lines. Number of dates of sampling for the long-term results is given in parentheses.

**Figure 11:**
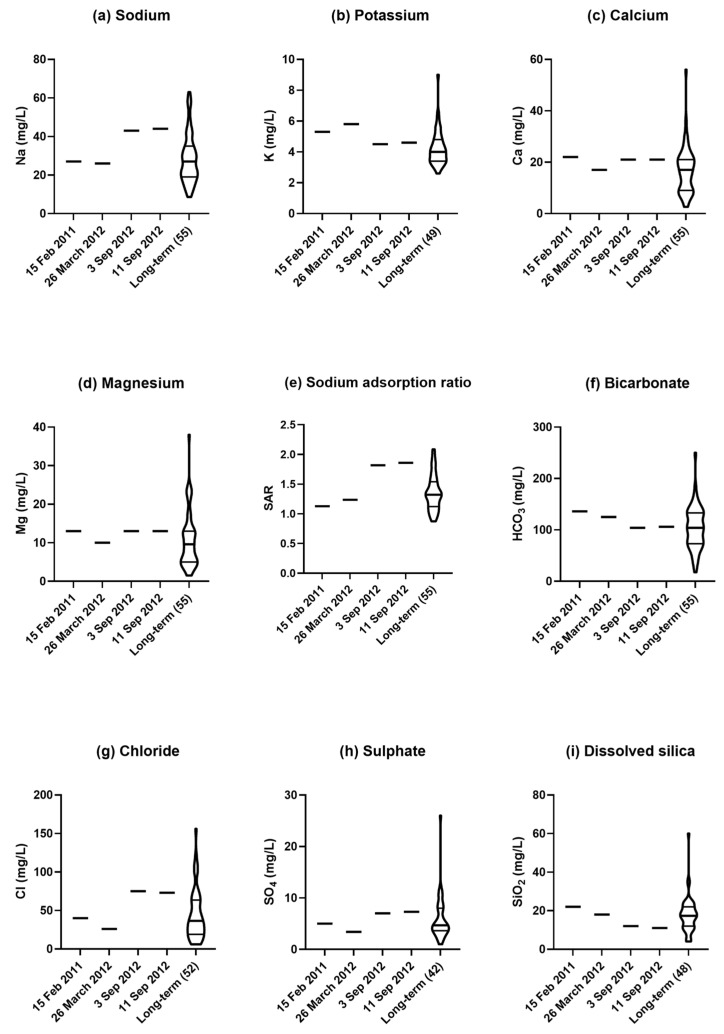
Major ions in water samples from the Cotswold Weir for four sampling dates, relevant to when the white encrustation was collected on 11 September 2012, in comparison with long-term results for the period from March 1971 to August 2019 [13]: (**a**) sodium; (**b**) potassium; (**c**) calcium; (**d**) magnesium; (**e**) sodium adsorption ratio; (**f**) bicarbonate; (**g**) chloride; (**h**) sulphate; and (**i**) dissolved silica. Each violin plot shows the smoothed frequency distribution of values for the trait of interest from long-term sampling, with the median indicated by the heavy horizontal line and first (lower) and third (upper) quartiles indicated by lighter horizontal lines. Number of dates of sampling for the long-term results is given in parentheses.

**Figure 12:**
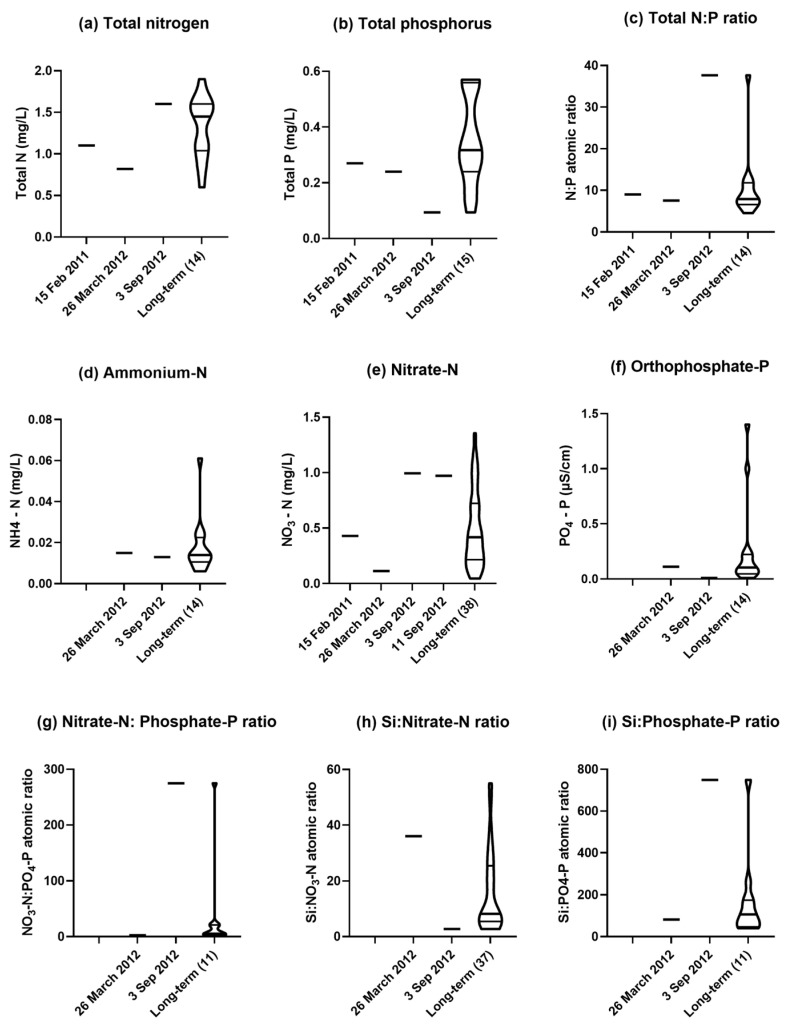
Nutrient concentrations in water samples from the Cotswold Weir for four sampling dates, relevant to when the white encrustation was collected on 11 September 2012, in comparison with long-term results for the period from March 1971 to August 2019 [13]: (**a**) total nitrogen; (**b**) total phosphorus; (**c**) total nitrogen/phosphorus atomic ratio; (**d**) ammonium-nitrogen; (**e**) nitrate-nitrogen; (**f**) orthophosphate-phosphorus; (**g**) nitrate-nitrogen/orthophosphate-phosphorus atomic ratio; (**h**) dissolved silica-silicon/nitrate-nitrogen atomic ratio; and (**i**) dissolved silica-silicon/orthophosphate-phosphorus atomic ratio. Each violin plot shows the smoothed frequency distribution of values for the trait of interest from long-term sampling, with the median indicated by the heavy horizontal line and first (lower) and third (upper) quartiles indicated by lighter horizontal lines. Number of dates of sampling for the long-term results is given in parentheses.

Results of analyses of water samples from the Cotswold Weir in the years 2011–2012 are given in Figs 10–12 in comparison with long-term results for samples from the site since 1971. The sampling date of 15 February 2011 was soon after the record floods had occurred in the river. The sampling date of 26 March 2012 was ~6 months before the white coating was observed on the weir wall, and the 3 and 11 September 2012 sampling dates were just before and at the time the white encrustation was collected, respectively.

The water temperature measured in 2012, ranged from 23 °C on 26 March (in Autumn) to 14 °C on 3 September (in early Spring) (Figure 10a). Turbidity of the water was relatively elevated in the third quarter on 15 February 2011, following the floods of that year, but in all samples in 2012, it was close to the first (lower) quartile (Figure 10b). Dissolved oxygen in March of 2012 was 8.7% in the fourth quarter and in September was 6.8% close to the long-term median (Figure 10c). Electrical conductivity was close to the long-term median in March, but it was much higher in the fourth quarter in September (Figure 10d). The pH ranged from 8.0 in the fourth quarter in March 2012 to 7.6 in the third quarter in September (Figure 10e). Similarly, total alkalinity (expressed in units of calcium carbonate) ranged from 104 mg/L near the third quartile in March to 86 mg/L in September, close to the long-term median (Figure 10f).

Among the major cations, sodium concentration in March 2012 (26 mg/L) was near the long-term median, but in September (43 mg/L) it was high in the fourth quarter (Figure 11a). Potassium concentration was in the fourth quarter in March 2012 (5.3 mg/L) and near the third quartile in September (4.5 mg/L) (Figure 11b). Both calcium (Figure 11c) and magnesium (Figure 11d) were close to their median values in March 2012 and somewhat higher near the third quartile in September. These changes in the balance of these cations are reflected in the sodium adsorption ratio, which was in the second quarter in March 2012, but high in the fourth quarter in September (Figure 11e).

Among the major anions, bicarbonate in March 2012 was at the third quartile (125 mg/L), but lower in September (104 mg/L) closer to the long-term median (Figure 11f). The pattern with chloride was different from that of bicarbonate, but similar to that of sodium with the value for chloride in March (26 mg/L) in the second quarter, whereas in September (75 mg/L) it was in the fourth quarter (Figure 12g). Sulphate showed a somewhat similar trend to chloride with a lower value in March (3.4 mg/L) in the first quarter than in September (7 mg/L) near the third quartile (Figure 11h). Dissolved silica showed a similar pattern to bicarbonate with a value in March (18 mg/L) in the third quarter falling in September (11 mg/L) to lie near the first quartile (Figure 11i).

Total nitrogen in the water was low in March 2012 in the first quarter, but high near the third quartile in September (Figure 12a). Total phosphorus was in the second quarter in March 2012, but at the minimum in September (Figure 12b). These divergent values were reflected in the total nitrogen/phosphorus atomic ratio with a value in September 2012 that was at the maximum (Figure 12c). Ammonium-nitrogen was near the median in both March and September (Figure 12d). Nitrate-nitrogen was in the first quarter in March 2012, but in the fourth quarter in September (Figure 12e). Orthophosphate-phosphorus was near the median in March 2012 but at the minimum in September (Figure 12f). The nitrate-nitrogen/orthophosphate-phosphorus atomic ratio was near the minimum in March 2012 but at the maximum in September (Figure 12g). The dissolved silicon/nitrate-nitrogen atomic ratio was high in the fourth quarter in March, but at the minimum in September 2012 (Figure 12h). The dissolved silicon/orthophosphate-phosphorus atomic ratio was 81.7 in the first quarter in March but at a maximum of 749 in September 2012 (Figure 12i).

The concentrations of dissolved and total metals recorded at Cotswold Weir for the September 2012 sampling are presented in Table 1. The dissolved metals complement the sodium, potassium, calcium and magnesium that are presented in Figs 1a–d. The highest concentrations of dissolved metals were strontium and barium. The highest concentrations of total metals were of aluminium and iron. The default guideline values for the 95% level of species protection that are presented in Table 1 are concentrations of dissolved metals and metalloids that if exceeded indicate potential changes in the biota of slightly to moderately disturbed freshwater systems [14].

Levels of organic molecules potentially related to mining activities were below the detection limits for phenols (<0.25 µg/L), polycyclic aromatic hydrocarbons (PAH) (<0.01 µg/L), and benzene, toluene, ethylbenzene and xylenes (BTEX) compounds (<0.2 µg/L).

## 3. Discussion

The previously unrecorded appearance of a white substance on the weir walls of the Condamine River caused concern among townspeople and the rural community that the water was contaminated, that it posed a health risk to humans and domestic animals, and was caused by mining activities. It is clear that the vitreous white coating on the wall of the Cotswold Weir was an accumulation of the frustules of dead diatoms. The material that was removed from the wall in flakes was the dried remnants of an epilithic periphyton (biofilm) that had developed on the concrete weir wall, consisting of filamentous algae with pennate and filamentous centric diatoms, together with amorphous material composed of clay particles and partly decomposed organic matter. The periphyton had likely developed on the wall during prolonged river flows, following record-breaking rainfall and flood events in late 2010 and early 2011, and the subsequent continual flow of water over the wall. A conducive period for the development of the periphyton on the weir wall, and the blooming of the diatoms, had been followed by a dry period with no water flowing over the weir side wall such that the diatoms had died, leaving their frustules at the surface. Correct diagnosis of the white substance as the equivalent of diatomaceous earth, instead of deposits of other chemicals, was important to allay fears of serious issues for human and animal health with water drawn from the river.

Understanding the nature of this white substance, should it reoccur, is important. Also important is to understand the biology of the diatoms and the environmental conditions that led to the bloom in case it is indicative of adverse trends in the river that require mitigation. The question arises as to how the diatoms managed to grow and remain in place on the near vertical weir wall as water flowed over it. The ability of diatoms to attach to surfaces in flowing freshwater was clearly revealed in the study of the formation of diatom biofilms on polished stainless-steel surfaces immersed for 6 months in the Oise River, a tributary of the Seine River in the north of France [15]. From environmental scanning electron micrographs, eight pennate diatom species were identified, including five species with mucilage attachments to the surface, three species motile on secreted mucilage including one *Nitzschia* sp., and one centric diatom species, namely *M. varians*, the same species identified in the white encrustation in our study. Mature biofilms formed on surfaces in freshwater consist of biological consortia of photoautotrophs (mainly microalgae including diatoms and cyanobacteria), and heterotrophs (bacteria and fungi), in a matrix of extracellular polymeric substances, which are important in keeping individual organisms in place in flowing water and in the functioning of the microbial community [16]. Thus, it is apparent that continual water flow would allow attachment of diatoms to the vertical wall of the weir and the extracellular polymeric substances produced would have kept them in place against the flow of water over the wall.

When a temporary stream, subject to the alternation of wet and dry hydrological phases, stops flowing, biofilms can suffer extreme desiccation and heating with a consequent large loss of cell densities and biomass of both algae and bacteria. Diatoms in such biofilms are more sensitive to desiccation than some other algae that have thicker cell walls [16]. Thus, once water stopped flowing over the Cotswold Weir side wall the diatoms in the biofilm would have been subject to desiccation and heating. This appears to have completely killed the biofilm diatoms as indicated by their non-recovery when flakes were incubated in water in the laboratory, even though a filamentous green alga was recovered. When water recommences flowing in an intermittent stream, the re-establishment of biofilms that have been decimated in the dry period can be from organisms that survived in situ as resting structures, and/or have survived in an upstream pool of water and then been transported in the rejuvenated stream to recolonize the site of the former biofilm. Some diatoms, including *Nitzschia* spp., are very efficient in colonising new substrata [17]. Thus, for this rare occurrence of the diatoms to predominate in the periphyton on the weir wall, conditions must have been suitable for inocula of the diatoms to be transported there from the weir pool followed by an extended period of water flow of suitable composition for them to bloom.

Diatoms are an important part of primary photosynthetic production in most aquatic systems and a key component underpinning food chains for aquatic macroinvertebrates and vertebrates. As such, diatoms are regarded as beneficial, and this rare bloom of diatoms is unlikely to have been problematic like previous blooms of toxic species of cyanobacteria in the Murray–Darling River system [18,19]. However, it could be important to understand why diatoms bloomed to such an extent in the Condamine River and whether this is signalling water quality changes in the river that could have adverse impacts in the future. Comparisons can be drawn from the study of the benthic diatom flora on cobbles in the upper reaches of the economically important Great Fish River of Eastern Cape Province in South Africa [11]. That river, like the Condamine River, runs through a semi-arid area with agricultural enterprises and is subject to flooding at irregular intervals. Dominant diatom taxa were mostly those considered to be pollution-tolerant. There were significant correlations of diatom species abundance with pH (sample range 7.5–9.4), nitrate-nitrogen (0.021–0.489 mg/L), electrical conductivity (120–1321 µS/cm), ammonium–nitrogen (0–0.9 mg/L), and calcium carbonate (28–214 mg/L). The main drivers affecting diatom community composition were electrical conductivity and nitrate-nitrogen, followed by pH and orthophosphate-phosphorus (range 0.02–0.638 mg/L). It was concluded that the river diatom community had been impacted by decades of agricultural activity, including irrigated cropping and dairying, leading to eutrophication and salinization. Among five ‘pollution tolerant’ species identified in that study were *N. palea* and *E. subminiscula* [11], species that were also present in the encrustation on the Cotswold Weir wall in our investigation. Holmes and Taylor [11] stated that *N. palea* was present at all their sampling sites in the Great Fish River, probably because of its ‘affinity for water of higher electrical conductivity levels’.

Consideration of the ecological requirements of diatoms, and specifically of the species identified in the encrustation on the Cotswold Weir wall, may shed light on why they bloomed. Different diatom species can have different growth requirements of pH, osmotic concentration, and inorganic nutrients. Descriptions in the literature provide information on the occurrence, preferred habitats, and water quality requirements and tolerances of various diatom species. All species identified from the Cotswold Weir wall are described as cosmopolitan [20]. The dominant diatom species on the weir wall, *Nitzschia palea*, is widespread in fresh waters, typically inhabiting the benthos of freshwater ponds and watercourses, and also soils [21]. The species is eurybiontic being able to tolerate a wide range of environments [21], including ‘heavily organically polluted waters’, moderate to high electrolyte concentrations, or heavy metal contamination, and to occur in both lotic and lentic waterways [12,20]. Ecological conditions for other species of diatoms identified in the encrustation from the Cotswold Weir wall have been described [20] as follows: (i) *Luticola mutica*: common in brackish conditions and in waters that are prone to drying out; (ii) *Eolimna subminiscula*: common in electrolyte-rich, strongly polluted rivers and streams; (iii) *Navicula viridula* var. *rostellata*: a eutrophic species, tolerant of critical levels of pollution; (iv) *Craticula cuspidata*: an epipelic species occurring in eutrophic waters with moderate to high electrolyte content, extending into brackish waters that may tolerate critical to very heavy pollution; and (v) *Melosira varians*: found in both the benthos and plankton, becoming particularly abundant in eutrophic, occasionally slightly brackish waters. Thus, the collective descriptions of the species of diatoms identified in the encrustation of the Cotswold Weir wall indicate communal tolerance of eutrophic and moderately saline conditions.

Since all the diatom species in the encrustation have been described as tolerant of eutrophic conditions, what does the chemical analysis of the water from the Condamine Weir leading up to the diatom bloom reveal in this regard? The total nitrogen concentrations of the Cotswold Weir water in 2012, which ranged from 820 µg nitrogen/L in March to 1600 µg nitrogen/L in September, would class it as mesotrophic, while the total phosphorus concentrations, which ranged from 240 µg phosphorus/L in March to 94 µg phosphorus/L in September would class it as eutrophic [22]. Much of the total nitrogen and total phosphorus causing increased eutrophication is in organic compounds bound to clay particles eroded from the land, along with additional inputs of decomposing organic matter of plant and animal origin transported into the river during floods. During this period the nitrogen/phosphorus atomic ratio changed from 7.6 (considered nitrogen limiting for algal growth) to 37.6 (considered phosphorus limiting for algal growth), and this ratio would have passed through 16, the value considered optimum for algal growth [22]. Thus, the Cotswold Weir water could be considered mesotrophic to eutrophic and conducive for algal growth, including for the eutrophic tolerant diatom species identified in the encrustation.

Most of the diatom species present in the encrustation are described as tolerant of mild salinity or elevated electrical conductivity The Condamine River is subject to fast-flowing conditions after high rainfall and slow-flowing conditions for much of the time. During periods of rapid flow with high volumes of water, the turbidity of the river water increases due to eroded soil particles in suspension, and the electrical conductivity decreases due to dilution by the large volumes of water entering the river system. During periods of low flow, the turbidity of the water decreases in weir pools and natural ponds due to sedimentation, and the electrical conductivity increases due to the concentration of salts through evaporation [4,6]. Although the concentration of salt in river water during a flood can be lower, the quantities of salt leached out of the soil and collected from other surface waters that enter the river system can lead to longer-term salinization of the river water. These contrasting effects were evident between the water samples from the Cotswold Weir in March 2012 during a period of high flow in the river, and in September 2012 during low flow. Between these dates, conditions in the river had been conducive for the diatoms to bloom. Although water in the Cotswold Weir would not be considered saline, the electrical conductivity and sodium and chloride concentrations were at historic high values in September 2012. This may have been associated with the entry of salts into the river system from diffuse and point sources during the record floods with subsequent concentration in the river through evaporation. However, there was not a uniform relative increase in the concentrations of different ions in the water. The results showed that sodium and chloride were close to the maximum recorded values for the Weir water in September 2012, and sodium was relatively more concentrated than calcium and magnesium as indicated by the higher SAR. Although SAR was the highest recorded from the Cotswold Weir, it was still below the threshold value of three that indicates the water is excessively sodic and problematic for irrigation because of the dispersion of clay in soils [23]. Overall, it would seem that the mild level of salinity, with increased sodium and chloride, in the river water favoured the growth of the diatom species identified here. Increasing salinization of inland waters, including rivers like the Condamine in semi-arid areas, is a worldwide problem [24], and continued monitoring is required to recognise and prevent adverse trends towards excessive salinization of the Condamine River.

Diatoms, in contrast to other microalgae and cyanobacteria, require dissolved silica for cell wall production. An average composition of diatoms is given by the Redfield-Brzezinski atomic ratio for silicon/nitrogen/phosphorus of 15:16:1 [25]. Also, the dissolved silicon/nitrate-nitrogen ratio (or inverted as the nitrogen/silicon ratio) has been used as an indicator of water conditions favouring diatom growth [26]. The dissolved silica concentration and the dissolved silicon/nitrate-nitrogen ratio in the Cotswold Weir water were both high in March 2012 (18 and 36, respectively), and fell to lower levels in September 2012 (12 and 2.7, respectively). A similar trend occurred with bicarbonate, which was high in March and lower in September, unlike the trend with other anions, particularly chloride. Bicarbonate can be used as carbon for photoautotrophic growth by *N. palea* and other diatom species [27]. The nitrate-nitrogen/orthophosphate-phosphorus ratio and the dissolved silicon/orthophosphate-phosphorus ratios in September 2012 were both high, which would have provided a competitive advantage for diatoms, because they are superior competitors for phosphate over other microalgae and cyanobacteria [26,28]. Thus, the levels of silica in the water were such to favour diatom growth. Dissolved silica levels had probably increased following the heavy rains with overland flow bringing eroded clay minerals and silica-containing grass residues into the river. Subsoil drainage into the river may also have contributed to dissolved silica levels (A. Biggs, pers. comm.).

The concentrations of all dissolved metals and metalloids in the Cotswold Weir water were below the respective default guideline values for protection of aquatic life in moderately disturbed freshwater systems [14], except for copper, which was just above the guideline value of 1.4 µg/L. Heavy metal pollution of rivers can cause deformities in periphytic diatom frustules, and also can change the species’ composition and abundance [9,29]. Microalgae, in general, are sensitive to copper concentrations, and although there would appear to be no major urban or industrial sources for copper pollution in the catchment, levels of copper in the Condamine River should be monitored.

A bloom of diatoms in the Condamine River is a rare event that has not been previously recorded, although all the cosmopolitan species identified would be part of the normal diatom flora of the river. The water conditions favoured the diatom bloom and this could be a natural phenomenon as part of the extremes of the hydrological cycle of the river, or it may have been exacerbated by anthropogenic effects from agricultural production and/or mining in the Condamine River catchment changing the water chemistry to favour diatom growth. Continued monitoring of the chemistry and biology of the river system is required to maintain a watch on its ecological health.

## 4. Materials and Methods

### 4.1. Macroscopic and Microscopic Examination of the White Flakes

The white coating on the weir wall naturally fractured into flakes. About 30 flakes were removed manually from random positions on the weir wall on 11 September 2012, and placed in a polythene sample bag. On return to the laboratory, the size of the flakes and the appearance of their top and underside were recorded. The thickness of the flakes was measured with a cover-slip screw micrometre (Leitz Australia, Brisbane). The surface of the flakes was observed under a stereo microscope (SZM model, Olympus, Tokyo, Japan) with incident light at magnifications up to ×100. Drops of dilute hydrochloric acid were placed on the upper surface of flakes while observing the reaction under the stereo microscope to check effervescence indicative of the presence of carbonates. Scrapings were taken of the white surface, mounted in water on microscope slides, and observed at magnifications up to ×1000 using differential interference contrast microscopy with a Vanox microscope (Olympus, Tokyo, Japan).

### 4.2. Illuminated Incubation of the Flakes in Water

Flakes (irregular shapes ~2.5 cm across) were placed in 250 mL glass jars, covered with ~2 cm of either tap water or deionized water (three replicates of each), and placed in the light from a window protected from direct sunlight. Microscope slides were made of the green material that appeared after 1 to 4 days of incubation and examined by brightfield and differential interference contrast microscopy at magnifications up to ×400. Slides of the dark underside of the flakes were also made and examined microscopically.

### 4.3. Scanning Electron Microscopy of the Flakes

Images of the undisturbed surface of the white substance were obtained with scanning electron microscopes (SEMs). Samples of the white flakes were adhered with a carbon conductive tab to an aluminium stub and gold coated with a sputter coater (Leica EM SCD005; Leica Microsystems, North Ryde, Australia). The gold coating was 10 nm thick and produced with programme settings of working distance = 5 cm, time = 75 s, and current = 30 mA. Scanning electron micrographs were obtained with a FEI Quanta 200 SEM (FEI Company, Hillsboro, OR, USA) operated under high vacuum at 10 kV with a tungsten filament and working distances of 7.5–4.8 mm. Further scanning electron micrographs were obtained using an ultra-high resolution field emission SEM (Zeiss Sigma; Carl Zeiss, North Ryde, Australia), operated under high vacuum at 10 kV, with field emission filament and Everhart–Thornley Detector (ETD), and working distances of 7.5–4.8 mm.

### 4.4. Physical and Chemical Analyses of the River Water

Water was sampled from the Cotswold Weir on the day the white flakes were collected. Standard protocols for collecting, handling and storage of water samples for physical and chemical analyses were followed [30,31]. In order to obtain a well-mixed water sample from the weir pool near the wall, a 5 L weighted container at the end of a long rope was immersed mid-stream and three samples were taken, the first to wash a 10 L bucket and the next two to fill it. From the water mixed in this bucket, sub-samples were poured into the sample containers supplied by Queensland Health, Forensic and Scientific Services (QHFSS).

Water samples for general analyses were collected in 1 L plastic bottles. Other samples for analysis of total metals were acidified with 2.5 mL of concentrated nitric acid added to a 250 mL bottle of water to displace any metals bound to suspended material in turbid water. Samples for analysis of dissolved metals were first passed through a syringe fitted with a Minisart 0.45 µm High Flow syringe filter into 100 mL bottles then acidified with 1 mL of concentrated nitric acid. Samples for BTEX were poured into two 40 mL bottles capped instantly, and for PAH and phenols into two brown glass 1 L bottles. Electrical conductivity, pH and turbidity were determined on-site using a LaMotte Tracer Pocketester (LaMotte, Chestertown, MD, USA) and a turbidity tube.

Comprehensive chemical analyses were carried out at the QHFSS laboratories, Coopers Plains, Brisbane, using standard laboratory procedures [32]. These included analyses for major cations (sodium, potassium, calcium, magnesium), major anions (bicarbonate, chloride, sulphate), nutrients (total nitrogen, total phosphorus, ammonium, nitrate, orthophosphate), dissolved silica, pH, electrical conductivity and turbidity, plus analyses for dissolved and total concentrations of 23 metals and metalloids (listed in Table 1). Analyses were also conducted for phenols associated with coal, BTEX found in petroleum products, and PAH present in coal. A database of physical and chemical analyses of water taken at 0.1 m depth from the Cotswold Weir periodically since March 1971 [13] was summarised to obtain violin plots of distributions and long-term values for medians and quartiles of the various properties using GraphPad Prism [33].

Sodium adsorption ratio (SAR) was calculated using the following formula with Na+, Ca++ and Mg++ ionic concentrations (expressed in milliequivalents/L):

SAR = Na/√(0.5(Ca + Mg))

The nitrogen/phosphorus ratio (atomic) was calculated from total nitrogen and total phosphorus concentrations [22]. Other nutrient atomic ratios [26] of (a) nitrate-nitrogen/orthophosphate-phosphorus; (b) dissolved silica-silicon/nitrate-nitrogen; and (c) dissolved silica-silicon/phosphate-phosphorus were also calculated.

### 4.5. Assessing Preceding Water Flows over the Cotswold Weir

The Cotswold Weir side wall, where the white encrustation occurred, is 4.5 m high, whereas the central section of the wall that allows water to flow at lower river levels is 4.29 m high (K. Klaasen, pers. comm.). The Queensland Department of Natural Resources, Mines and Energy has maintained a stream flow gauging station (number 422325A) at the Cotswold Weir to record the volume and level of water flow in the river daily [13]. The days when water would have flowed over the side wall or over the lower central section of the Cotswold Weir during 2010–2012 were determined from these data of daily river levels.

## 5. Conclusions

Correct diagnosis of the white encrustation that occurred on the weir wall, as the mass aggregation of diatom frustules, alleviated concerns among townspeople and the rural community about alternative causes, such as salt or carbonate deposition, or other chemicals arising from mining activities in the Condamine Basin. The tightly packed frustules of dead diatoms formed the surface of a dehydrated periphyton that had developed during protracted water flows after record rainfall and flooding. Neither the siliceous frustules nor the previously living diatoms pose risks for human or animal health. Five species of pennate diatoms and one centric diatom identified in the encrustation are cosmopolitan and tolerant of eutrophic and mildly saline waters. Chemical analysis of the water indicated it was mesotrophic for total nitrogen and eutrophic for total phosphorus, with high atomic ratios for nitrate-nitrogen/orthophosphate-phosphorus, dissolved silica-silicon/nitrate- nitrogen and dissolved silica-silicon/orthophosphate phosphorus that would favour diatom growth. This new understanding of the diatomaceous nature of this white coating and its formation, with a protracted river flow allowing the blooming of diatoms, followed by flow cessation and consequent death with bleaching of the diatoms, allays community speculation and excessive concern for the health of the river and for people and animals drinking from it.

## Figures and Tables

**Table 1:** Dissolved and total metals and metalloids from Cotswold Weir, 11 September 2012, with guideline values of dissolved metals for comparison.

Metal	Dissolved (µg/L)	Total (µg/L)	95% Species Protection Default Guideline Value (µg/L Dissolved) ^a^
Aluminium	4 ± 0.3 ^b^	570 ± 43	55 (for pH > 6.5)
Antimony	<0.1 ^c^	<0.1	Sb(III) 9
Arsenic	0.4 ± 0.02	0.5 ± 0.03	As(III) 24; As(V) 13
Barium	72 ± 4.9	83 ± 5.6	NA ^d^
Beryllium	<0.1	<0.1	0.13 (indicative)
Boron	56 ± 7.8	55 ± 7.7	370
Cadmium	<0.1	<0.1	0.2
Chromium	0.1 ± 0.01	0.7 ± 0.07	Cr(VI) 1
Cobalt	0.2 ± 0.01	0.9 ± 0.07	1.4
Copper	2 ± 0.15	3 ± 0.23	1.4
Iron	9 ± 0.77	590 ± 50	NA
Lead	<0.1	0.3 ± 0.03	3.4
Manganese	0.9 ± 0.08	95 ± 8.0	1900
Molybdenum	0.5 ± 0.03	0.4 ± 0.03	34
Nickel	2.7 ± 0.18	3.5 ± 0.24	11
Selenium	<1.0	<1	11
Silver	<1	<1	0.05
Strontium	260 ± 19.0	260 ± 19.0	NA
Thallium	<0.1	<0.1	0.03
Titanium	2 ± 0.18	7 ± 0.64	NA
Uranium	0.1 ± 0.01	<0.2	0.5
Vanadium	5.5 ± 0.59	7.2 ± 0.77	6
Zinc	<1	2 ± 0.18	8

^a^ Default guideline values are dissolved concentrations in slightly to moderately disturbed freshwater ecosystems [14]. ^b^ Concentration ± measurement uncertainty. ^c^ < signifies concentration below the limit of recording. ^d^ NA = not available.

## Data Availability

The data are available on reasonable request of the authors.
